# Ontologies as the semantic bridge between artificial intelligence and healthcare

**DOI:** 10.3389/fdgth.2025.1668385

**Published:** 2025-08-29

**Authors:** Radha Ambalavanan, R Sterling Snead, Julia Marczika, Gideon Towett, Alex Malioukis, Mercy Mbogori-Kairichi

**Affiliations:** Research Department, The Self Research Institute, Broken Arrow, OK, United States

**Keywords:** ontologies, semantic interoperability, artificial intelligence, healthcare integration, clinical decision support, natural language processing, ethical AI, multi-modal data integration

## Abstract

**Background:**

Ontologies serve as a foundational bridge between artificial intelligence (AI) and healthcare, enabling structured knowledge frameworks that enhance data interoperability, clinical decision support, and precision medicine.

**Objective:**

This perspective aims to highlight the essential role of ontologies in enabling adaptive, interoperable frameworks that evolve with technological and medical advances to support personalized, accurate, and globally connected healthcare solutions.

**Methods:**

This perspective is based on a targeted literature exploration conducted across PubMed, Scopus, and Google Scholar, prioritizing studies published between 2010 and 2025 and including earlier seminal works where necessary to provide historical context, focusing on ontology-driven AI applications in healthcare. Sources were selected for their relevance to semantic integration, interoperability, and interdisciplinary applicability.

**Results:**

Through the standardization of medical concepts, relationships, and terminologies, ontologies enable semantic integration across diverse healthcare datasets, including clinical, genomic, and phenotypic data. They also address challenges such as fragmented data and inconsistent terminologies. This semantic clarity supports AI applications in clinical decision support, predictive analytics, natural language processing (NLP), and patient-specific disease modeling.

**Conclusions:**

Despite their transformative potential, ontology integration faces significant challenges, including computational complexity, scalability, and semantic mismatches across evolving international standards, such as SNOMED CT and HL7 FHIR. Ethical concerns, particularly around data privacy, informed consent, and algorithmic bias, also require careful consideration. To address these challenges, this perspective outlines strategies including adaptive ontology models, robust governance frameworks, and AI-assisted ontology management techniques. Together, these approaches aim to support personalized, accurate, and globally interoperable healthcare systems.

## 1 Introduction

Modern healthcare systems continue to face challenges related to fragmented data, lack of standardized vocabularies, and poor semantic alignment. Ontologies, structured frameworks that define standardized concepts and relationships within a domain (1). They enable consistent data interpretation, support automated reasoning, and enhance clinical decision-making. By facilitating semantic interoperability across systems, ontologies allow AI models and healthcare applications to integrate and act upon complex clinical, genomic, and phenotypic data (2–4).

Ontologies, such as SNOMED CT (Systematized Nomenclature of Medicine – Clinical Terms) and HL7 FHIR (Fast Healthcare Interoperability Resources), developed by Health Level Seven International (HL7), serve as foundational tools by standardizing medical concepts, terminologies, and data structures (5). These unified frameworks support tailored patient-specific healthcare solutions, improving clinical outcomes and patient experience. They can enhance the efficacy of Clinical Decision Support Systems (CDSS) by providing structured knowledge representation and facilitating rule-based reasoning (6). Ontologies also support personalization of healthcare by adapting intervention plans to individual needs and uncovering hidden comorbidities (7).

By enabling interoperability across Electronic Health Records (EHRs), ontologies facilitate seamless collaboration between healthcare providers and AI systems. They drive advancements in precision medicine and patient-centered care by standardizing medical terminologies and ensuring consistent data exchange. Furthermore, ontologies integrate diverse data sources by aligning terminologies like SNOMED CT, LOINC (Logical Observation Identifiers Names and Codes), and ICD-11 (International Classification of Diseases, 11th Revision) with real-world applications, demonstrating their potential to enhance data-driven decision-making and AI-driven analytics. These attributes make transformative innovations in both AI and healthcare systems (5, 8, 9).

Ontologies offer a solution by bridging artificial intelligence (AI) and healthcare (1). Despite their value, significant challenges remain, particularly in addressing fragmented datasets, lack of standardization, and ethical concerns like data privacy, and adaptive ontology models offer promising solutions by supporting real-time updates, promoting ethical governance, and allowing culturally sensitive customization (10). Through semantic integration and fostering patient-centered design, ontologies are poised to bridge gaps in healthcare systems and advance AI-driven solutions.

The evolution of ontologies, initially rooted in statistical classifications and formal logic, has progressively transformed to address the multifaceted demands of modern healthcare. From supporting clinical operations and reimbursement processes to enabling translational research and reducing errors, improving efficiency, and driving biomedical discovery, ontologies have demonstrated their adaptability and growing importance. Their structural evolution is guided by diverse application areas, allowing them to meet the complex requirements of contemporary healthcare. These applications are particularly significant in supporting international data harmonization and ensuring consistent interpretation across healthcare systems. Currently, ontologies form the foundation for integrating vast biomedical datasets, supporting sophisticated data science inferencing, and enabling advanced analytics and decision-making tools that drive innovation in healthcare systems (5).

By providing a systematic approach to knowledge representation, ontologies enable AI systems to process biomedical data with greater precision and reliability. Their flexibility to adapt to new data and evolving standards reinforces their role in advancing healthcare and AI systems (11, 12).

In this perspective, we highlight the need for adaptive ontology models that evolve with real-time data and emerging standards to support precision medicine, promote ethical AI integration, and address key gaps in semantic interoperability. We outline the historical evolution of ontologies, mechanisms enabling interoperability, real-world applications, and future directions for their development in healthcare innovation. This perspective synthesizes key literature on ontology-driven AI in healthcare, outlines current challenges in semantic interoperability, and proposes a roadmap for advancing adaptive, ethically grounded, and globally interoperable ontology models. It also highlights the reciprocal relationship between ontologies and AI, showing how ontologies enhance AI capabilities while AI methods drive ontology evolution and automation.

## 2 Methods

We conducted a targeted literature exploration to identify key advancements, current challenges, and gaps in ontology-driven applications of artificial intelligence in healthcare. Searches were performed across PubMed, Scopus, and Google Scholar using combinations of keywords aligned with the article's thematic focus, including “ontology,” “artificial intelligence,” “interoperability,” “semantic integration,” “clinical decision support,” “personalized medicine,” “natural language processing,” and “ethical AI.” These were combined with Boolean operators such as (ontology OR ontologies) AND (“artificial intelligence”) AND (“interoperability” OR “semantic integration” OR “clinical decision support” OR “personalized medicine” OR “natural language processing” OR “ethical AI”).

We prioritized peer reviewed publications published between 2010 and 2025 that addressed the development or application of ontologies in artificial intelligence, healthcare interoperability, and related interdisciplinary domains, while excluding sources lacking direct relevance to biomedical or clinical applications. We also included earlier seminal works published before 2010 when they were considered foundational or essential for understanding the historical evolution of ontology-driven AI applications.

This manuscript was intentionally developed as a perspective rather than a scoping or systematic review. Given the interdisciplinary and conceptual nature of the topic, which spans technical, biomedical, and policy domains, conducting a comprehensive systematic review was beyond the intended scope of this work. Accordingly, this study applies a conceptual grouping of recurring themes to synthesize key insights, emerging trends, and challenges, with the goal of informing future research and development in ontology-driven, AI enabled healthcare systems.

## 3 Ontologies

### 3.1 Historical context

Ontologies originated in philosophy, where early thinkers like Aristotle developed taxonomies to classify knowledge. As computer science advanced, ontologies evolved into structured systems for representing domain knowledge. Gruber (1993) defined them as “explicit specifications of a conceptualization” (13), while Guarino (1998) emphasized their value in organizing domain-specific knowledge (14). The introduction of formal ontology languages like RDF (Resource Description Framework) and OWL (Web Ontology Language) (15–17) enabled ontologies to support semantic integration across fragmented datasets in dynamic, multi-user environments (18).

A useful distinction exists between formal ontologies, which provide general upper-level concepts across domains, and application ontologies, which are specifically designed for particular domains such as oncology or cardiology. This distinction is especially relevant in AI and healthcare, where both abstraction and domain specificity are essential.

To remain relevant in fast-evolving fields like AI and healthcare, ontologies must continually adapt to new data, contexts, and standards. During the COVID-19 pandemic, ontologies rapidly incorporated new clinical terminologies and concepts such as “COVID-19-associated pneumonia” or “long COVID” to support data interoperability, surveillance, and research (19). Evolutionary processes, such as alignment, versioning, and meaning negotiation, are critical in distributed healthcare ecosystems where real-time collaboration and interoperability are required (20, 21). Especially in AI-driven healthcare, ontology evolution supports adaptive systems that can integrate diverse knowledge sources, maintain semantic consistency, and ensure accurate decision-making (22, 23).

### 3.2 Semantic integration

Ontologies play a pivotal role in enabling semantic integration by providing a structured and hierarchical approach to organizing data. They establish a shared vocabulary, enabling diverse systems to communicate seamlessly and interpret information consistently. This hierarchical organization supports advanced reasoning and inference, allowing for the transformation of raw data into actionable insights. To demonstrate their real-world relevance and technical sophistication, the following subsections explore practical applications of semantic integration and the underlying mechanisms that enable its implementation across AI-driven healthcare systems.

#### 3.2.1 Permission to reuse and copyright

In modern healthcare, ontologies are instrumental in bridging diverse datasets such as clinical, genomic, and environmental data into unified and interpretable frameworks. Their structured vocabularies enable systems like the Gene Ontology (GO) and the Foundational Model of Anatomy (FMA) to annotate and cross-reference biological and anatomical information, facilitating precision medicine and real-time clinical decision-making (24).

By aligning genomic, phenotypic, and clinical information, ontologies support diagnostic accuracy, treatment stratification, and predictive analytics in AI applications (25). These capabilities are reflected in practical applications such as the use of geospatial ontologies, which integrate location-based data to enhance public health planning during disease outbreaks or environmental exposures (26). These real-world applications highlight why semantic integration is essential, not only for linking datasets but also for enabling intelligent, adaptive, and context-aware healthcare systems.

#### 3.2.2 Underlying technical mechanisms

This semantic depth is powered by technical mechanisms such as hierarchical taxonomies, logical inference engines, and standardization frameworks. Taxonomies define relationships between broad and specific concepts, enabling structured reasoning and context-aware classification. Ontology-based engines validate data consistency and deduce new knowledge from complex, multimodal datasets (24, 27, 28).

Foundational ontologies such as DOLCE (Descriptive Ontology for Linguistic and Cognitive Engineering) and BFO (Basic Formal Ontology) facilitate alignment across domain-specific ontologies, resolving semantic discrepancies and supporting cross-domain compatibility. BFO, for instance, provides a unifying framework that enables consistent mapping of clinical concepts like “disease onset” in SNOMED CT with biological processes in the Gene Ontology (GO) (25).

Standards such as RDF and OWL support interoperability by enabling shared concept mapping, while SPARQL (SPARQL Protocol and RDF Query Language) improves query performance across large biomedical datasets (29). These technical tools enable inter-ontology reasoning, allowing AI systems to synthesize knowledge across clinical, genomic, and environmental domains, thereby advancing translational research and supporting tailored care strategies (30).

### 3.3 Ontology development and maintenance

Ontology development is a systematic and iterative process essential for building robust frameworks in complex domains, including healthcare and AI. Effective ontology development includes clearly defined stages: design, validation, alignment, versioning and updates, enrichment, and ontology evolution. These stages collectively address challenges like fragmented datasets, inconsistent terminologies, and evolving domain-specific requirements, ensuring that ontologies remain adaptable and relevant. Such modularity is essential in healthcare AI, where domain knowledge and terminologies rapidly evolve, making this design process vital for AI–healthcare convergence.

#### 3.3.1 Design phase

The design phase begins by identifying domain-specific requirements through consultation with domain experts, existing documentation, and user needs analysis. This phase is crucial to ensure that the ontology accurately represents real-world contexts and aligns with domain-specific standards (31). Techniques like Ontology-Driven Conceptual Modeling (ODCM) provide theoretical guidance, enabling structured representations and conceptual clarity through languages like OntoUML (32).

#### 3.3.2 Validation methodologies

Robust validation ensures that ontologies reliably support AI accuracy in healthcare applications. Competency Questions (CQs) test whether an ontology can answer clinically relevant queries, such as linking symptoms to diagnoses or treatments, which is essential for decision support tools. Metrics like consistency and cohesion assess structural quality, while tools like Protégé automate reasoning and error detection (33). These methods help AI systems make accurate predictions, especially in domains like oncology and cardiology where precise semantic alignment is critical (34).

#### 3.3.3 Alignment

Ontology alignment harmonizes various domain-specific ontologies to facilitate seamless interoperability and consistent data exchange. Semantic mapping techniques link different ontologies, establishing compatibility and coherent data interpretation across healthcare and AI systems. Foundational ontologies including DOLCE and BFO assist this alignment by providing universal frameworks for categorization and ensuring data consistency (35).

#### 3.3.4 Versioning and updates

Ontologies must continually evolve to reflect new data, updated standards, and emerging knowledge. Robust versioning systems are essential for managing changes, maintaining backward compatibility, and ensuring long-term usability. One such method, the Ontology Metadata Vocabulary for Ontology Data Management (ON-ODM), helps track changes across versions by documenting metadata such as authorship, updates, and dependencies. This allows ontology frameworks to adapt to evolving domain needs while preserving accuracy and relevance over time (36).

#### 3.3.5 Enrichment techniques

Enrichment involves refining ontologies by adding new concepts, relationships, and data properties, which enhances their completeness and contextual relevance. Artificial intelligence, particularly NLP and text-mining techniques, accelerates this process by scanning large volumes of biomedical literature, clinical notes, or research databases to identify emerging terms or relationships that may be missing from existing ontologies.

The BioPortal Annotator uses NLP to map free-text inputs to existing biomedical ontologies, enabling automatic detection of relevant concepts and suggesting updates based on current literature. This dramatically improves the speed and accuracy of ontology maintenance by reducing manual curation efforts and ensuring timely integration of new knowledge (37).

Such AI-driven enrichment ensures that ontologies stay current and aligned with evolving healthcare needs, supporting high-quality AI applications and clinical decision support systems (CDSS).

#### 3.3.6 Ontology evolution in collaborative environments

Ontology evolution is essential in collaborative and distributed environments like the Semantic Web, where ontologies dynamically co-evolve alongside user communities. The key processes include meaning negotiation, alignment of multiple organizational ontologies, and management of contextual dependencies. These iterative evolution processes maintain ontology robustness, facilitating real-time knowledge integration essential for modern AI-driven healthcare solutions (18).

### 3.4 Broader applications of ontologies

Ontologies are increasingly applied across diverse fields such as bioinformatics, environmental science, robotics, education, and interdisciplinary research. By providing structured vocabularies and semantic frameworks, they enable data integration, automation, and personalized decision-making. This versatility makes ontologies powerful tools for advancing innovation, interoperability, and knowledge-sharing across domains. This cross-domain adaptability underscores the foundational role of ontologies in fostering innovation in complex, data-driven environments like healthcare AI. Table 1 summarizes ontology applications across domains, highlighting their purposes and implementation scenarios.

**Table 1:** Cross-domain applications of ontologies.

Domain	Ontology	Purpose	Application scenarios
Bioinformatics and genomics	Gene Ontology (GO) (38)	Standardize biological terms for functional genomics and gene annotation.	Cross-species gene function comparison, annotation of omics data.
	Sequence Ontology (SO) (39)	Support genomic annotation and integration of omics datasets.	Protein function curation in GOA, alignment of clinical and genomic data.
Environmental sciences	Environment Ontology (ENVO) (40)	Standardize environmental data for geospatial and ecological analysis.	Climate change studies, natural resource monitoring, pollution tracking.
	ENVO in Spatial Data Infrastructures (SDIs) (41)	Enable semantic interoperability in spatial data infrastructures.	Disaster management, environmental policymaking through unified data integration.
Robotics and automation	Ontology for Collaborative Robotics and Adaptation (OCRA) (42)	Support adaptive robotics and real-time human-robot collaboration.	Industrial robotics, dynamic plan adaptation, safety reasoning.
	A Semantic Technology Approach for Knowledge Management in E-Government (ASTRO Project Ontology) (43)	Enhance interoperability and task planning in robotic systems.	Smart environments, service orchestration, autonomous decision support.
Education and e-Learning	Learning Object Metadata Ontology (LOM) (44)	Personalize digital learning, structure tutoring systems, and enable interoperability across LMS.	Adaptive assessments, personalized feedback, real-time learning analytics.
Neuroscience	Cognitive Atlas (45)	Maps mental functions to tasks for use in cognitive neuroscience research.	Task annotation in fMRI studies, construction of cognitive concept models, linking behavior with brain function.
	Neuroscience Information Framework Standardized Ontologies (NIFSTD) (46)	Provides structured vocabularies for brain anatomy, functions, disorders, and experimental methods.	Integration of neuroimaging datasets across platforms, neuroscience data standardization, and brain atlas mappings.
Cross-disciplinary collaboration	European Health Data Space (EHDS) and European Open Science Cloud - Life Sciences (EOSC-Life) Frameworks (47, 48)	Facilitate interdisciplinary collaboration and governance in health and life sciences data.	Continental-scale health data governance, FAIR data reuse in life sciences.

## 4 AI and ontology applications in healthcare

By addressing challenges linked to fragmented datasets, disconnected systems, and inconsistent medical data representation, ontologies provide a structured framework that enables improved data integration, semantic clarity, and scalable AI-driven solutions in healthcare. They provide a structured framework for organizing diverse healthcare information, ensuring semantic interoperability across clinical applications. By standardizing how data is defined and interpreted, ontologies improve clinical decision-making and support AI models that rely on consistent, high-quality input. Ontologies bridge technology and human-centered care, becoming foundational tools in advancing precision medicine, healthcare automation, and intelligent diagnostics. Their ability to standardize medical data into machine-readable formats empowers healthcare providers and AI-driven systems to derive actionable insights, enhancing patient outcomes and fostering healthcare innovation. Across domains such as EHRs, genomics, pharmacology, and geospatial analytics, ontologies ensure semantic consistency and enable scalable, intelligent applications in real-world care. The following subsections highlight key ontology applications in healthcare AI, from EHR interoperability to population-level predictions. Thus, ontologies are crucial in healthcare and AI.

### 4.1 Enhancing EHR functionality through ontologies

Ontologies integrated into EHR systems improve data clarity, reduce fragmentation, and enhance care coordination. They go beyond standard interoperability by embedding meaning and relationships directly into data, enabling smarter clinical workflows. Ontology-driven systems and EHR-integrated analytics have demonstrated tangible impacts on clinical efficiency. For instance, Rizzoli Orthopaedic Institute reported a 30% reduction in hospitalizations and over 60% fewer imaging tests by using advanced analytics to manage hereditary bone disorders. Similarly, Kaiser Permanente used EHR-linked analytics to minimize unnecessary antibiotic use in newborns, significantly lowering both side effects and resource utilization. These examples reflect how integrated data systems can optimize patient care and reduce redundant interventions without compromising quality (49). They also enable context-aware decision support by aligning terminologies like SNOMED CT and LOINC with patient records, flagging interactions or missing clinical documentation (50).

Frameworks like openEHR and ISO 13606 use a dual model approach that includes reference models for structure and archetype models for clinical semantics. These models, supported by HL7 FHIR, form the foundation of ontology-aligned systems that can interpret and exchange structured clinical data consistently (51). The canonical ontology model adds a shared conceptual layer that enables different EHR architectures to speak a common language, allowing automated reasoning and intelligent data exchange (52). NHS Digital in the UK utilizes such models to ensure that care records follow patients across systems, reducing duplication and improving continuity (53).

### 4.2 Clinical decision support and AI-driven diagnostics

Ontologies enhance CDSS by structuring medical knowledge into logically connected frameworks that AI systems can reason over. They enable context-aware AI to connect symptoms, diagnoses, and treatments through explicit semantic links, thereby improving diagnostic accuracy and personalized care.

Ontology-driven systems support intelligent automation and reasoning by aligning terminologies across diverse data sources, such as SNOMED CT, LOINC, and HL7 FHIR. These integrations reduce errors, identify care gaps, and guide clinicians in selecting optimal interventions. By improving semantic clarity and ensuring real-time data alignment, ontologies foster clinical workflows that are both evidence-based and adaptive to patient-specific contexts (54).

Explainable AI (XAI) further builds trust in these systems by showing how decisions are made. This transparency is crucial in clinical settings, where trust and accountability are essential. Addressing data quality and model bias through semantic structuring also increases clinician confidence in AI-driven diagnostics (55).

### 4.3 Genomic and phenotypic data integration

The Human Phenotype Ontology (HPO) enables AI to connect clinical traits to genetic causes, supporting early diagnosis and precision treatment. A widely adopted example is its use in rare disease diagnostics, where HPO annotations help clinicians prioritize likely genetic causes based on patient-reported symptoms (56).

Whole-genome sequencing (WGS) platforms combined with ontology-based annotation frameworks improve variant interpretation and treatment decisions (57). By standardizing phenotypic descriptors, HPO supports data harmonization across studies, reducing annotation variability and enabling AI systems to draw consistent insights from clinical-genomic data (34).

### 4.4 Pharmacogenomics and AI-driven treatment recommendations

Ontology frameworks such as PharmGKB (Pharmacogenomics Knowledgebase) and Drug Ontology (DrON) encode drug-gene relationships in structured vocabularies, enabling AI systems to process pharmacogenomic knowledge. These ontologies help AI systems predict optimal drug dosing and identify potential adverse reactions (58).

A real-world application is CPIC (Clinical Pharmacogenetics Implementation Consortium), which uses ontology-based rules to generate treatment recommendations based on genetic test results. By integrating ontologies with EHR and genomic data, CPIC delivers precise, evidence-based recommendations tailored to a patient's genetic profile (34, 59).

### 4.5 Patient-specific disease modeling

Ontologies support personalized disease modeling by capturing complex relationships among clinical symptoms, genetic variants, and disease progression patterns. Specialized disease-specific ontologies such as the Disease Ontology (DO) and Orphanet Rare Disease Ontology (ORDO) offer structured vocabularies that allow AI systems to assess patient risk profiles more precisely. These ontologies define standardized terms for disease subtypes, stages, and comorbidities, which enhances individualized prognosis modeling.

In clinical practice, ontology-enriched platforms for neurodegenerative diseases integrate imaging, genomic, and behavioral data to detect early signs of disease progression. For example, Parkinson's disease modeling systems use ontology-guided inputs to identify subtle symptom changes over time, improving staging accuracy and treatment response predictions (34, 58).

### 4.6 Predictive analytics and population health management

Ontology-based predictive analytics enable healthcare systems to anticipate risk and guide preventive interventions. By aligning structured genomic, lifestyle, and environmental data, these models support early detection of chronic diseases such as diabetes or heart failure. For instance, Mount Sinai Health System in New York implemented an ontology-driven predictive model to identify patients at high risk of 30-day readmission, enabling early follow-up and significantly reducing avoidable hospitalizations (55).

In public health, ontologies drive disease surveillance by standardizing how conditions, locations, and risk factors are coded. This allows for faster outbreak detection and supports targeted health campaigns. Clinicians benefit from real-time alerts and visual summaries that support early interventions and risk mitigation strategies (58).

### 4.7 Geospatial databases in healthcare

Geospatial ontologies bring structure to location-linked health data, allowing integration of patient demographics, environmental exposures, and disease patterns. These models support GeoAI, which applies AI to spatial health analytics for decision-making in resource planning, disaster response, and disease tracking.

A notable example is the use of Virtual Knowledge Graphs (VKGs) in regional COVID-19 tracking platforms, which integrated mobility data, infection rates, and healthcare capacity to guide containment strategies (26). Ontology-driven GIS platforms enhance map-based decision tools, offering public health teams spatial insights that are both contextualized and updated in real time (60).

## 5 Ontology-enabled applications in healthcare AI

Ontologies empower healthcare AI by offering explicit, logic-based knowledge structures that improve data interoperability, model accuracy, and informed decision-making. They standardize medical concepts, align terminologies, and create meaningful relationships among data points, enabling AI systems to integrate and reason over diverse datasets such as EHRs, genomic profiles, and public health records. Beyond clinical information, ontologies enhance geospatial reasoning and population-level health analysis through spatial semantics. Additionally, ontologies are pivotal in enabling secure data governance, supporting privacy preservation, secure data sharing and patient privacy, thus substantially advancing precision medicine and healthcare innovation.

### 5.1 Knowledge representation and AI reasoning

In oncology, AI systems supported by ontologies can analyze pathology reports, match patient profiles to treatment guidelines, and flag inconsistencies for clinician review. These real-world applications rely on structured frameworks for representing medical knowledge. Ontologies allow AI to interpret clinical data accurately by embedding formal logic into how conditions, symptoms, and procedures are related.

According to Hoehndorf et al. (2017), knowledge graphs and semantic models built on ontologies significantly improve consistency checks, classification, and deductive inference, allowing AI systems to uncover hidden patterns within medical datasets (61). RDF-based semantic knowledge graphs coupled with inference engines can help AI enforce data consistency and support reasoning across clinical records.

As emphasized by Chudasama et al. (2023), these knowledge structures improve AI interpretability, enabling context-aware recommendations and bridging gaps between clinician knowledge and AI-generated insights (62). By grounding AI decisions in formal ontologies, models become more transparent, traceable, and aligned with medical logic. This structure facilitates rule-based diagnostics and generates clinically relevant explanations, ensuring trust in AI-driven decision-making. Ontologies enhance AI reasoning by structuring clinical knowledge into consistent, computable logic.

### 5.2 Natural language processing (NLP) in healthcare

Ontology-driven NLP transforms unstructured clinical text into standardized, machine-readable formats that enhance AI performance and consistency. Unlike traditional AI systems that typically work with structured datasets, natural language processing (NLP) tools, particularly large language models (LLMs), are uniquely capable of extracting meaning from unstructured clinical text, such as discharge summaries, referral letters, and observational notes, and converting it into computable, interoperable formats.

Ontology-based NLP enhances semantic interoperability and reduces ambiguity in medical terminology by aligning extracted concepts with structured vocabularies like SNOMED CT and UMLS. A typical case is the term “cold,” which may refer to either a viral illness or a sensation of low temperature. Ontology-driven systems resolve this by evaluating the clinical context and linking the term to the appropriate concept in SNOMED CT (63–65). This is essential for processing physician notes, discharge documents, and patient communications (65, 66). Real-world implementations include AI-powered chatbots and voice-enabled assistants that use ontology-driven NLP to interpret symptoms, retrieve relevant medical facts, and document clinical encounters in real time, which reduces the manual entry burden for providers (62, 67). Fareedi et al. (2025) emphasized the role of ontologies in managing dialogue context and intent recognition in medical chatbot systems. By focusing on language-specific ambiguities and terminology alignment, ontology-driven NLP allows downstream AI tools to function more effectively, supporting real-time documentation, entity recognition, and decision-making across varied clinical environments (67). Ontology-driven NLP transforms clinical text into structured data, enabling real-time AI support.

### 5.3 Predictive analytics and machine learning

Ontologies improve predictive analytics and machine learning by structuring biomedical data, enhancing diagnostic precision, optimizing early detection, and personalizing treatments. By standardizing inputs across clinical, demographic, and behavioral datasets, ontologies reduce data noise and improve the reliability of predictive models (68, 69). Structured ontology-driven analytics identify high-risk patients, enable early interventions, and optimize population health management (70). Their role extends to model explainability and bias reduction by embedding clinically validated relationships into machine learning pipelines (71). Standardized ontologies improve predictive accuracy and support early, personalized interventions.

### 5.4 Explainable AI and trustworthy systems

Explainability is crucial for fostering trust, transparency, and regulatory compliance in AI-driven healthcare. Ontologies enhance explainability by structuring knowledge representations that increase model interpretability, aligning AI decisions closely with human reasoning. Neuro-symbolic AI approaches integrate ontologies with deep learning, enhancing both accuracy and interpretability by embedding formal domain knowledge (72). Semantic reasoning, supported by OWL2 description logics and SWRL (Semantic Web Rule Language), allows AI systems to perform automated inference consistent with domain expertise, further improving explainability (73, 74). Additionally, structured ontology-driven methodologies reduce biases and enhance fairness across diverse patient populations. Provenance tracking and semantic knowledge graphs support auditability and accountability, thus reinforcing trust in AI-generated clinical recommendations and regulatory compliance (75, 76).

Ethical and regulatory standards, such as the EU Artificial Intelligence Act, highlight the necessity for interpretable, accountable AI models in healthcare. Ontologies support this need by encoding explicit rules and traceable data relationships, which facilitate transparency in automated predictions. This capability aligns with legal frameworks such as the General Data Protection Regulation (GDPR) and the Health Insurance Portability and Accountability Act (HIPAA) (77, 78). Ontologies strengthen explainability, fairness, and accountability in AI-driven healthcare decisions by providing traceable, interpretable logic that supports compliance with evolving regulatory standards.

### 5.5 Multi-modal data integration

Ontology-driven AI significantly enhances multi-modal data integration by systematically organizing heterogeneous healthcare datasets, including clinical notes, genomic sequences, imaging scans, and phenotypic observations, into unified, interoperable formats. Ontologies act as the semantic glue that aligns these data types by mapping them to shared vocabularies and hierarchical relationships. For example, ontological standards such as SNOMED CT and HPO enable consistent tagging of diagnoses and phenotypes, while structured imaging metadata is integrated using standard terminologies. This allows AI models to analyze these modalities together for richer clinical insights. A real-world application can be seen in oncology platforms, where SNOMED CT-coded diagnoses, radiology findings, and HPO-based phenotype annotations are combined to build comprehensive patient profiles that support AI-driven treatment recommendations (73, 79). FAIR principles further improve the accessibility and reusability of multi-modal datasets, supporting robust decision-making that aligns with data governance standards (77). Semantic knowledge graphs derived from these ontologies strengthen integration by bridging clinical, imaging, and molecular data. This approach drives innovation in precision medicine and personalized healthcare. As multi-modal AI continues to evolve, ontology-driven frameworks are likely to remain essential for enabling real-time, adaptive, and precision-guided healthcare interventions. Semantic ontologies unify diverse datasets, powering adaptive, precision-guided clinical insights.

## 6 Challenges and limitations

The development and implementation of ontologies in healthcare face a range of practical and conceptual challenges. These include issues related to scalability, interoperability, domain knowledge representation, ethics, and version control. Table 2 summarizes these key challenges, illustrating real-world examples and outlining evidence-based strategies to address them.

**Table 2:** Key ontology challenges and strategies in healthcare.

Domain	Limitation description	Real-world case	Suggested tools and strategies
Complexity and scalability	Large-scale healthcare ontologies require significant computational resources and can impact system responsiveness when scaling. Scaling without disrupting existing logic or introducing inconsistencies is challenging (80).	Integrating disease symptoms and treatment pathways across multiple hospital systems requires scalable ontology design (81).	Use frameworks like DOGMA to separate core ontology elements from domain-specific customizations for better scalability (82).
Interoperability and standardization issues	Incompatibilities in structured data formats, rapidly evolving terminologies, and fragmented domain standards disrupt seamless data exchange across healthcare systems. Integrating well-defined patient-generated data with established clinical standards such as HL7 FHIR and SNOMED CT remains a complex and ongoing challenge (83, 84).	Integrating wearable device data with EHRs poses a major challenge in harmonizing patient-generated and clinical records (85).	Employ shared canonical models, apply Apache Spark for real-time ontology harmonization, and promote unified terminologies through global collaboration. A component-based framework and self-sovereign identity (SSI) enhance alignment, semantic interoperability, and secure data exchange (86, 87).
Fuzzy ontology in semantic interoperability	Uncertain or probabilistic clinical inputs, such as vague symptoms, incomplete diagnoses, or imprecise terminology, are poorly handled by deterministic ontologies. This limitation affects semantic interoperability across distributed systems (88).	A fuzzy ontological model was developed to align heterogeneous healthcare terminologies and enable better data integration across SNOMED CT, HL7 FHIR, and LOINC (89).	Apply fuzzy logic to enhance semantic matching, resolve ambiguity in terminology, and support flexible interoperability across evolving healthcare standards (89).
Domain expertise and knowledge representation	Difficulty in capturing tacit medical knowledge and achieving expert consensus leads to ambiguity and inconsistency in ontology design. Variability arises from differing clinical interpretations (90, 91).	The Cancer Care Ontario project combined physician input with structured clinical knowledge to enhance pathways in oncology, including breast cancer treatment planning and follow-up care coordination (92).	Implement AI-supported frameworks to distinguish between objective data and expert judgments, ensuring flexibility and consistency (93).
Ethical and privacy concerns in healthcare ontologies	Data privacy risks, algorithmic bias, and consent management issues pose challenges in ethically deploying ontologies in healthcare (94).	Unfair treatment recommendations due to biased data highlight the need for fairness-aware machine learning (95).	Adopt SSI frameworks, strong encryption, bias detection tools, and XAI methods for transparent and fair systems (87).
Maintenance and versioning challenges	Frequent updates risk redundancy and semantic conflicts without structured governance and version control mechanisms (96).	SNOMED CT's biannual update process ensures backward compatibility through community review and validation pipelines (97).	Use structured governance, validation tools, and stakeholder collaboration to manage updates while maintaining compatibility (98).

## 7 Enhancing healthcare ontologies for global integration

The integration of healthcare ontologies is essential for achieving semantic interoperability, data standardization, and cross-border collaboration. Leveraging AI-driven automation, modular architectures, and governance frameworks supports scalable, adaptive, and internationally aligned healthcare systems. Ensuring multi-domain compatibility and collaborative knowledge representation will drive innovation in precision medicine, clinical decision support, and biomedical research.

### 7.1 Collaborative ontology development

Developing robust healthcare ontologies requires collaboration among clinicians, researchers, informaticians, and policymakers, ensuring accurate and globally meaningful knowledge representation. International initiatives, including SNOMED CT adoption, HL7 FHIR integration, and multinational ontology projects, play a crucial role in harmonizing healthcare data globally (99). Open-source platforms like BioPortal, OpenEHR, and Linked Open Data (LOD) further facilitate data accessibility, transparency, and innovation, fostering collaborative knowledge sharing (100).

Cross-institutional alignment, enabled by advanced methods like Multiview Incomplete Knowledge Graph Integration (MIKGI), significantly enhances interoperability across diverse healthcare environments (101). However, persistent barriers such as regional healthcare policies, inconsistent terminology mappings, and large-scale accuracy management continue to pose significant challenges. Overcoming these requires internationally coordinated governance frameworks, automated ontology translation tools, and continuous policy alignment.

### 7.2 AI-driven ontology automation

Advancements in AI and NLP enhance ontology development by significantly reducing manual efforts in ontology curation, enabling automated updates, error detection, and efficient semantic alignment. Traditional manual ontology curation is resource-intensive and prone to errors. NLP-driven approaches, including ontology learning from clinical texts and named entity recognition (NER), automate concept extraction, synonym identification, and hierarchical classification (102). AI-assisted tools employing deep learning, neural embeddings, and ontology alignment frameworks efficiently detect inconsistencies and improve semantic alignment (103). However, despite their promise, these AI-driven methods come with limitations. Models may generate hallucinated concepts, introduce misclassifications, or perpetuate bias from training datasets, especially in sensitive clinical contexts. Balancing automation with expert validation is therefore critical. AI-driven semantic mapping capabilities, when combined with human oversight, can enhance data harmonization while preserving accuracy and improving ontology adaptability to evolving medical knowledge (10). Future research should focus on developing adaptive ontology frameworks capable of autonomous evolution in response to emerging healthcare data demands. AI-assisted ontology learning approaches and automated semantic alignment frameworks are crucial for maintaining domain-specific adaptability and real-time update management.

### 7.3 Standardization for global interoperability

Standardized ontologies establish a structured foundation for data exchange, ensuring AI-driven healthcare solutions remain interoperable and efficient. Aligning healthcare ontologies with international standards such as HL7 FHIR, SNOMED CT, LOINC, and ICD supports consistent medical data representation and enables seamless data exchange across providers and research networks (104). However, key interoperability challenges persist, including semantic mismatches, hierarchical misalignments, and terminology inconsistencies between standards such as ICD-10, ICD-11, and SNOMED CT. These discrepancies complicate integration across domains and increase the risk of misinterpretation.

Efforts from global governance bodies such as HL7 International, ISO TC 215 Committee, WHO Digital Health Technical Advisory Group, and the Joint Initiative Council for Global Health Informatics Standardization are working to align ontologies through harmonized terminologies, formal data models, and coordinated updates. Advances in multi-view alignment, such as BERT-based models and graph neural networks, also improve semantic reconciliation and terminology mapping (105). Ontology standardization is of utmost importance as it ensures consistent data interpretation and exchange across various healthcare systems, regardless of the source or format. This harmonization promotes interoperability and facilitates compliance with dynamic regulatory frameworks, such as GDPR and HIPAA (106). AI-driven compliance monitoring and semantic reconciliation frameworks are essential to manage these complexities effectively. AI-enabled compliance tools and ontology-based reconciliation systems are expected to play an increasingly vital role in managing these complexities and maintaining global interoperability.

### 7.4 Scalability and adaptability of ontologies

As healthcare data grows in volume and complexity, ontology frameworks must scale efficiently and adapt to evolving medical knowledge. Static ontologies often fall short when faced with rapid developments in fields like genomics, pharmacology, and wearable health data. Modular ontology architectures, which use core structures extended through independent but compatible modules, support flexibility, reuse, and more efficient updates. For instance, Interface-Based Modular Ontology Formalism (IBF) helps tailor ontologies to specific clinical domains while maintaining overall coherence (107). In real-world settings, multi-domain integration that combines clinical records with omics and sensor data is essential for precision medicine. Projects that use FHIR-based ontologies and AI-driven reconciliation frameworks demonstrate how adaptable ontologies improve semantic interoperability and facilitate consistent data exchange across systems (108). However, maintaining continuous updates while ensuring backward compatibility remains a key challenge, especially when integrating legacy systems. Structured versioning protocols, AI-assisted change management, and automated ontology migration tools help address these issues (109). As a result, scalable and adaptable ontologies are critical not just for technical integration but also for enabling responsive, data-driven care models that improve outcomes across healthcare settings.

### 7.5 Limitations

This perspective reflects a targeted exploration of the literature rather than a comprehensive systematic review. The selection of sources was based on relevance to ontology-driven applications in AI-enabled healthcare, which may not fully represent all relevant literature, as studies outside the chosen databases (PubMed, Scopus, and Google Scholar) were not included. Although the primary focus was on studies published between 2010 and 2025, some earlier seminal works were included to provide historical context, which may slightly broaden the timeframe beyond the initial prioritization. Additionally, this work uses a conceptual grouping of recurring themes to synthesize insights, rather than applying quantitative synthesis or formal quality assessment, which may limit the reproducibility and generalizability of findings. These limitations are inherent to the perspective format, which aims to present key insights and guide future research directions rather than provide an exhaustive or statistically validated review.

## 8 Conclusion

Ontologies provide the structural backbone for integrating AI into healthcare by supporting semantic interoperability, enhancing decision-making, and enabling scalable, data-driven systems. As healthcare data grows in complexity, the importance of standardized, adaptable frameworks becomes increasingly clear. This perspective underscores the significance of ontologies in enabling AI-driven analytics, personalized care, and seamless data exchange while addressing key challenges such as standardization, governance, and scalability.

Looking ahead, research should prioritize the development of adaptive ontologies capable of evolving with changing healthcare demands and minimizing the burden of manual updates. Future research should also explore approaches that enhance privacy and prioritize patient-centered care in AI-driven systems. The synergy between ontologies and AI is vital for building transparent, explainable, and trustworthy healthcare systems. By fostering interdisciplinary collaboration and applying intelligent automation, ontologies can enable ethically grounded, scalable, and personalized healthcare innovations. Importantly, this perspective underscores the reciprocal relationship between ontologies and AI, highlighting how ontologies empower AI-driven applications while AI advances ontology evolution and maintenance.

## Data Availability

The original contributions presented in the study are included in the article/Supplementary Material, further inquiries can be directed to the corresponding author.
